# The Effect of Expanded Graphite Content on the Thermal Properties of Fatty Acid Composite Materials for Thermal Energy Storage

**DOI:** 10.3390/molecules29133146

**Published:** 2024-07-02

**Authors:** Dongyi Zhou, Shuaizhe Xiao, Yicai Liu

**Affiliations:** 1 School of Energy and Mechanical Engineering, Hunan University of Humanities, Science and Technology, Loudi 417000, China; 2 School of Mechanical and Energy Engineering, Shaoyang University, Shaoyang 422000, China; 19375383902@163.com; 3 School of Energy Science and Engineering, Central South University, Changsha 410083, China

**Keywords:** expanded graphite, thermal properties, composite phase-change materials, thermal energy storage

## Abstract

The mass content of expanded graphite (EG) in fatty acid/expanded graphite composite phase-change materials (CPCMs) affects their thermal properties. In this study, a series of capric–myristic acid/expanded graphite CPCMs with different EG mass content (1%, 3%, 5%, 8%, 12%, 16%, and 20%) were prepared. The adsorption performance effect of EG on the PCMs was observed and analyzed. The structure and thermal properties of the prepared CPCMs were characterized via scanning electron microscopy, differential scanning calorimetry, thermal conductivity measurements, and heat energy storage/release experiments. The results show that the minimum mass content of EG in the CPCMs is 7.6%. The phase-change temperature of the CPCMs is close to that of the PCMs, at around 19 °C. The latent heat of phase change is equivalent to that of the PCM at the corresponding mass content, and that of phase change with an EG mass content of 8% is 138.0 J/g. The CPCMs exhibit a large increase in thermal conductivity and a significant decrease in storage/release time as the expanded graphite mass content increases. The thermal conductivity of the CPCM with a mass content of 20% is 418.5% higher than that with a mass content of 5%. With an increase in the EG mass content in CPCMs, the heat transfer mainly transitions from phase-change heat transfer to thermal conductivity.

## 1. Introduction

Energy scarcity and environmental pollution are global challenges. The main means of solving the current energy crisis and environmental pollution problems are to save energy and rapidly develop renewable and clean energy, and thermal energy storage technology cannot be ignored in these measures [1]. Sensible energy storage, latent energy storage, and chemical energy storage are the three main types of thermal energy storage techniques [2]. Latent heat storage is the utilization of heat absorbed or released by energy storage materials during phase changes, and the amount of heat stored mainly depends on the latent heat of the phase change of the energy storage materials. A high energy storage density, minimal temperature fluctuations, strong material stability, and increased safety are some of the attributes of latent heat storage [3,4]. Therefore, it is widely used in building energy conservation, power peak shifting and valley filling, industrial waste heat and waste heat recovery, electronic device overheating protection, solar and geothermal energy utilization, and other thermal energy storage and temperature control fields [5,6,7]. At the core of latent heat energy storage are phase-change materials, and various inorganic, organic, and mixed phase-change materials have been studied in building energy conservation, such as paraffin [8,9], polyols [10,11], inorganic salts [12,13], and fatty acids [14,15]. Fatty acids have garnered significant interest because of their favorable phase transition temperature, high latent phase transition heat, lack of toxicity, corrosion, low undercooling, minimal or no volume change, excellent thermal stability, and wide-ranging raw material availability [16,17]. In addition, two or more fatty acids can form low-eutectic mixtures and obtain different phase-change temperatures to adapt to different temperature requirements in engineering practice. They are frequently utilized in low-temperature thermal energy storage systems, which include those used for building energy conservation and solar energy usage [18,19]. Although the thermal behavior of eutectic mixes of fatty acids is good, the thermal conductivity is low, and the material is more likely to leak out of the container when it undergoes a phase change from a solid to a liquid form. Therefore, it is necessary to mix and shape fatty acid eutectic mixtures, prevent leakage, and improve thermal conductivity. To create composite phase-change materials with good thermal conductivity, high-thermal-conductivity media are typically added to phase-change materials. Carbon materials (such as expanded graphite and carbon nanotubes) and metal particles and oxides are the most prevalent materials employed as high-thermal-conductivity media. Metal materials and metal oxides have excellent thermal conductivity; therefore, Fe, Cu, Al, Ag, Al_2_O_3_, Fe_2_O_3_, Fe_3_O_4_, C_u_O, MgO, TiO_2_, etc., are often used as thermal-conductivity-enhancing media for phase-change materials [20]. The addition of porous structural elements with high thermal conductivity to fatty acid phase-change materials is a technology that is currently being employed, and it not only improves the poor thermal conductivity of fatty acids but also stops fatty acid leakage during phase change [21,22,23,24]. Expanded graphite (EG) is a high-thermal-conductivity material with a porous structure and stable morphology at high temperatures, making it one of the most commonly used materials [25,26].

Zhu, H et al. prepared a lauric acid–stearic acid/EG (LA–SA/EG) CPCM using different mass fractions of LA–SA as PCMs and the addition of 10 wt.% EG to improve the thermal conductivity. The test results showed that, after the addition of EG, the thermal conductivity was nearly 10 times higher than before [27]. Wang, Z et al. also prepared an LA–SA/EG CPCM. Their results demonstrated that the heat storage and release effects of the heat storage device were optimal at an EG ratio of 10% weight percent and that the phase-change temperature and latent heat of the CPCM were stable when the EG content exceeded 5% [28]. Meng, X et al. used a eutectic mixture of CA, LA, and PA as PCMs to make fatty acid/expanded graphite CPCMs; as a result, the composite materials’ thermal conductivity was greatly increased [29]. Wen RL et al. prepared CA–LA/EP (capric acid–lauric acid/expanded property) and CA–LA/EVM (capric acid–lauric acid/expanded verticillite), which were predicted to have an expanded property and expanded verticillite, and added 5 wt.% expanded graphite to both PCMs. They found that the thermal conductivity of CA–LA/EP and CA–LA/EVM increased by 89.14% and 87.41%, respectively [30]. Luo, K. et al. used expanded graphite as a heat-conductive material, gas-phase silica (FS) as a carrier material, and LA–SA as a PCM to make lauric acid–stearic acid gas-phase silica expanded graphite (LA–SA–FS–EG). They compared this with an LA–SA–FS composite material without the addition of EG, and it was found that the thermal conductivity of LA–SA–FS–EG–7% containing 7% EG increased by 454%, the thermal storage rate increased by 46.1%, and the thermal release rate increased by 59.4% [31]. Fei, H et al. prepared a series of composite phase-change materials of capric acid–stearic acid octadecanol/expanded graphite (CSOD/EG), and they determined the optimal mass ratio of CSOD to be 81.9:9.1:9. The test results showed that CSOD was evenly breathed into porous EG without causing a chemical reaction and CSOD/EG exhibited good thermal reliability and chemical structural stability after 1000 cycles [32]. Fei, H et al. prepared MA–PA–TD/EG (myristic acid-palmitic acid-tetradecanol/expanded graphic) and MA–SA–LA/EG (myristic acid-stearic acid-lauric acid/expanded graphic CPCM), and the results showed that both CPCMs had good stability and were suitable for use in the field of building energy conservation [33]. Badenhorst, H believes that graphite materials can potentially alleviate the low-thermal-conductivity problem in phase-change materials (PCMs) when used for solar thermal energy storage, and the most economical option appears to be compressed expanded graphite composite materials [34]. Liu S et al. prepared a CA–SA/EG CPCM, and the results showed that the thermal conductivity of the CPCM with a 10% EG mass content was 3.28 times higher than that of the CA–SA PCM [35]. Ao, C et al. prepared SA–EG/EG CPCMs at different ratios, and the results showed that the CPCMs had a heat release rate 3.1 times that of SA and a heat storage rate 2.3 times that of SA [36]. Sari, A prepared PA/EG CPCMs with different mass contents (5%, 10%, 15%, and 20%), and the results showed that, when the mass content of EG was 20%, the thermal conductivity of the CPCM was 60 W/(m·k), which was 3.5 times that of PA [37]. The research on graphite or other materials as supporting materials in previous works and in this study is summarized in Table 1.

Numerous studies have shown that adding an appropriate amount of EG to fatty acid PCMs can significantly improve the thermal conductivity of composite materials, and composite materials have good thermal reliability and thermal cycling stability. However, there are few reports on the impact of the EG mass content on the thermal performance of CPCMs in systematic research. In this study, a series of fatty acid CPCMs with different EG mass contents (1%, 3%, 5%, 8%, 12%, 16%, and 20%) were prepared by taking capric–myristic acid/expanded graphite as an example. The minimum EG mass content in the CPCMs was determined using the diffusion–exudation circle method, and the structure and thermal properties of the prepared CPCMs were characterized. To verify the thermal conductivity of the composite materials, further tests to determine heat storage and release were conducted. This research represents an interesting background for the application of fatty acid phase-change materials in building energy-saving systems.

## 2. Materials and Methods

### 2.1. Materials

Shanghai Shanpu Chemical Co., Ltd., Shanghai, China, supplied the following acids: capric acid (CA, ≥98.5% purity), lauric acid (LA, ≥98.5% purity), myristic acid (MA, ≥98.5% purity), palmitic acid (PA, ≥98.5% purity), and stearic acid (SA, ≥98.5% purity). Table 2 displays the characteristics of the CA, LA, MA, PA, and SA. We bought expandable graphite from Qingdao Hengrunda Graphite Products Co, Ltd. in Qingdao, China. It had 350 meshes, a 100 mL/g expansion rate, a 99% carbon content, and a density of 1.1 g/cm^3^.

### 2.2. Preparation of Fatty Acid Binary Eutectic Mixtures/EG CPCMs

A specific volume of expandable graphite was subjected to a drying process in a vacuum drying oven. After drying, the expanded graphite could be obtained via high-temperature heating in a muffle furnace, with an expansion temperature of 900 °C and an expansion time of 30–50 s. The apparent density of the expanded graphite was estimated to be about 10 kg/m^3^, the pore diameter was 2–100 nm, and the specific pore surface area was about 30 m^2^/g. Binary low-eutectic mixtures of fatty acids were created by combining melt mixing and ultrasonic oscillation with the raw materials of the CA, LA, MA, PA, and SA [42,43].

The prepared binary low-eutectic mixture of fatty acids and expanded graphite was mixed in a beaker, thoroughly stirred, sealed with a thin film, and placed in a vacuum drying oven at 50 °C. The low-eutectic mixture of the binary fatty acid/EG composite phase-change energy storage material was obtained by heating the combination for 48 h, stirring it every 2 h, and cooling it to room temperature [44]. This process ensured that the mixture uniformly adsorbed EG.

### 2.3. Methods

The thermal properties of the CPCMs were measured by using a differential scanning calorimeter (DSC, NETSZCH 214Polyma, Selb, Germany) at a heating rate of 5 °C/min from 0 °C to 100 °C under a nitrogen atmosphere. The surface morphological structure of the CPCM samples was observed using a scanning electron microscope (SEM, phenom LE, Thermo Fisher Scientific, Waltham, MA, USA). The thermostability of the CPCM samples was investigated using thermogravimetric analysis (TGA, TA TGA5000IR, New Castle, DE, USA). The samples were heated at a rate of 10 °C/min, from 20 to 400 °C, in a nitrogen atmosphere, with an error of ±0.2%. A Fourier transform infrared spectrometer (FT–IR, Thermo Scientific Nicolet iS10, Waltham, MA, USA), operating in the 400–4000 cm^−1^ range with a resolution of 2 cm^−1^, was used to examine the material’s chemical structure.

The thermal conductivity of the CPCM samples was measured at room temperature using a DRE–III thermal conductivity tester (Xiangtan Xiangyi Instrument & Instrument Co., Ltd., Xiangtan, China) based on the transient plane heat source technique, with a measurement accuracy within ±3%. A dry sample and a probe were placed within a cake-shaped mold with a 3 cm diameter, and this was placed on a test bench after pressing it to the necessary density. After adjusting the settings based on the sample, the device was turned on to heat the probe. The software (Transient planar heat source method thermal conductivity testing system 2019.01) swiftly and precisely analyzed the sample, reported the results, and recorded the voltage and temperature increases over the measurement period.

**Figure 1:**
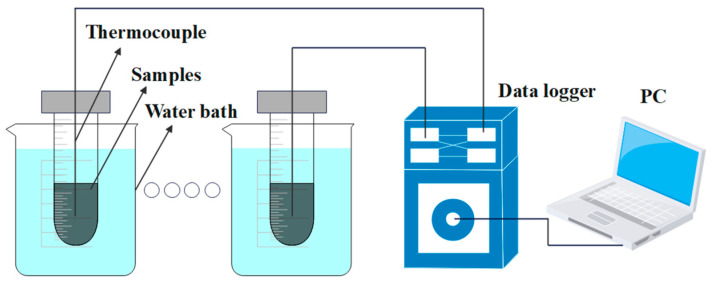
Experimental setup for heat energy storage/release tests.

Heat energy storage/release tests were carried out on the CPCM samples to confirm that the addition of EG improved their thermal conductivity. The experimental setup is shown in Figure 1. Temperature probes were placed into the center of the beaker with the same volume of CPCM. Then, the beakers were put into the same constant-temperature environment for a sufficient amount of time to achieve the set temperature at the center of the materials. The temperature was set to 42 °C for the heat energy release experiment and 12 °C for the heat energy storage experiment. Afterwards, the beakers were placed in a water bath to start the testing process. A steady temperature was maintained in the water bath throughout the testing procedure.

### 2.4. Experimental Uncertainty

There was a degree of uncertainty surrounding the experiment because of the measuring device’s precision. The uncertainty of the temperature measurement was assessed using the root-sum-square (RSS) approach [45,46]. Temperature variations were measured using a K-type thermocouple, which had an uncertainty of ±1.5 °C. The uncertainty of the temperature data logger was ±0.5 °C. The accuracy of the constant-temperature water bath was ±1.0 °C. The overall uncertainty *e_Temp_* of the temperature measurement system was calculated as follows:

(1) $$e_{Temp}=\sqrt{1.5^{2}+0.5^{2}+1^{2}}=1.87\;\text{°C}$$

## 3. Results and Discussion

### 3.1. The Adsorption Properties of EG on PCMs

**Figure 2:**
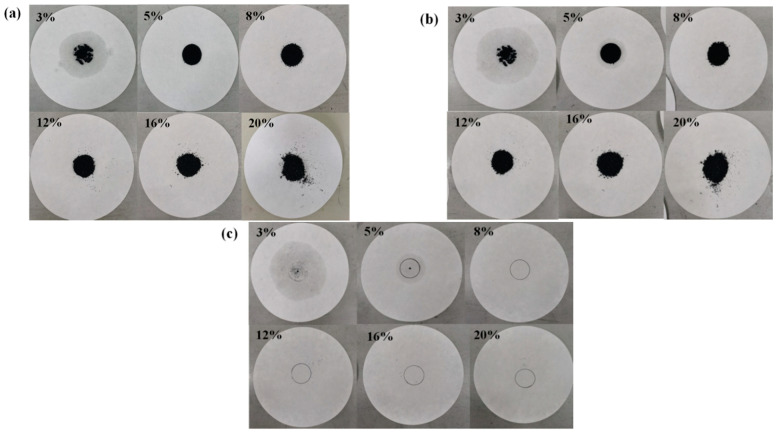
Photographs of CA–MA/EG CPCM samples with different EG mass contents before and after heat treatment. (**a**) Before heat treatment, (**b**) after heat treatment, (**c**) boiling off the material.

In fatty acid/expanded graphite CPCMs, fatty acids, as heat storage materials, are adsorbed in porous graphite, a support material. The higher the mass content of the heat storage material in the composite, the better the heat storage capacity of the CPCM. We propose a diffusion–exudation circle method to determine the adsorption characteristics of EG in PCMs [47]. Taking CA–MA/EG as an example, six groups of CA–MA/EG CPCMs with different EG mass contents (1%, 3%, 5%, 8%, 12%, 16%, and 20%) were prepared. The sample with a 1% EG mass content was obviously in a liquid form; hence, no heat treatment test was conducted. The results of the other six groups before and after heat treatment are depicted in Figure 2. Figure 2a shows an image before heat treatment, Figure 2b shows an image after heat treatment, and Figure 2c shows an image after boiling off the material. According to the method in Reference [47], a permeability stability evaluation was conducted, and the calculation results are shown in Table 2. In Figure 2, it can be observed that there was significant leakage in the samples with EG mass contents of 3% and 5%, and a large amount of the PCM had still not been adsorbed into the pores. This is because the adsorption capacity of the pores for the liquid PCM reached a saturation state. The samples with an EG mass content of 8% had almost no leakage, while the samples with an EG mass content of 10% or greater remained stable, without leakage. Based on the above analysis and the results in Table 3, it could be determined that the minimum EG mass content is around 8%. To ascertain the precise minimum mass content of EG in the CA–MA/EG CPCM, we prepared four samples with mass contents of 7.2%, 7.4%, 7.6%, and 7.8%, and we determined that the minimum mass content of EG in the CA–MA/EG CPCM was 7.6% by employing the aforementioned method [48].

### 3.2. Thermal Properties of the CPCMs

**Figure 3:**
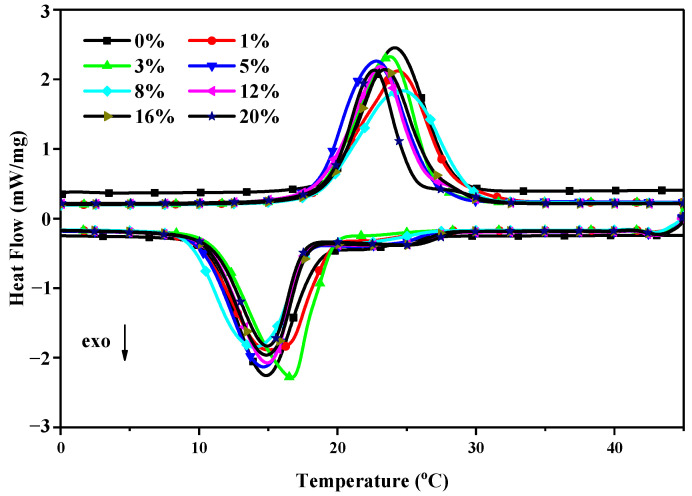
DSC curves of CA–MA and CA–MA/EG CPCMs with different EG mass contents.

**Figure 4:**
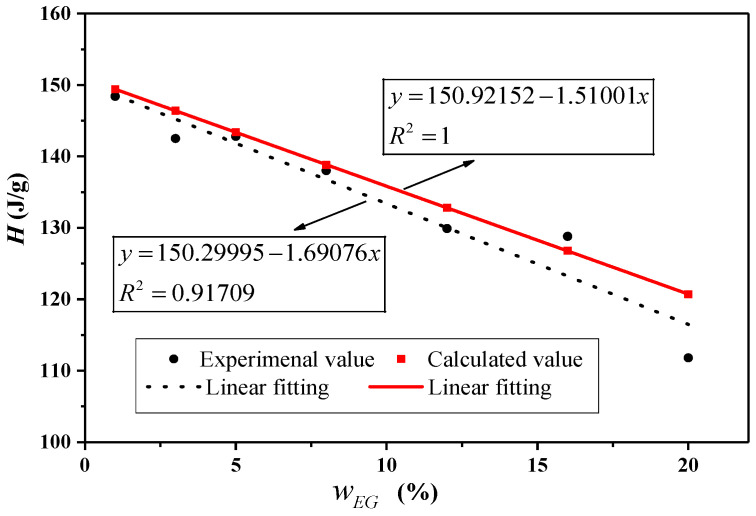
Comparison of the calculated and experimental values of phase transition latent heat for CA–MA/EG CPCMs with different EG mass contents.

Taking the CA–MA/EG CPCM as an example, the DSC curves of the CA–MA/EG CPCMs with different EG mass contents are shown in Figure 3, and the phase-change temperature and latent heat are shown in Table 4. It can be seen from the table that the phase-change temperature of the CPCMs with the addition of EG changes very little, indicating that the addition of EG has almost no effect on the phase-change temperature of CPCMs and that EG only plays a supporting role in the phase-change material. The latent heat of phase change can also be calculated using Equation (2). Table 4 and Figure 4 present the theoretical latent heat of the phase-change calculation findings for the CA–MA/EG CPCMs with different EG mass contents and compare them with the experimental test data.

(2) $$\Delta H_{CPCM}=\left(1-w\right)\Delta H_{PCM}$$

Here, $\Delta H_{CPCM}$ is the calculated latent heat of the CA–MA/EG CPCMs, *w* is the EG mass content in the CA–MA/EG CPCMs, and $\Delta H_{PCM}$ is the mass percentage of MA in the latent heat of CA–MA.

It can be seen in Table 3 and Figure 4 that the experimental data are very close to the theoretical values, with a small error, within 10%, indicating that the addition of EG has little effect on the latent heat of the CA–MA/EG CPCM. The latent heat of the CA–MA/EG CPCM decreases with the increase in the EG mass content, and it shows an almost-linear relationship. For example, when the mass content of EG is 20%, the latent heat of the sample is 109.8 J/g, while pure CA–MA has a latent heat of 150.9 J/g, with a ratio of approximately 73%, which is close to the mass fraction of CA–MA in the CPCM of 80%. This suggests that the adsorption of CA–MA in EG is only physical absorption and EG has little effect on the phase-change latent heat.

### 3.3. Microstructure of the CPCMs

**Figure 5:**
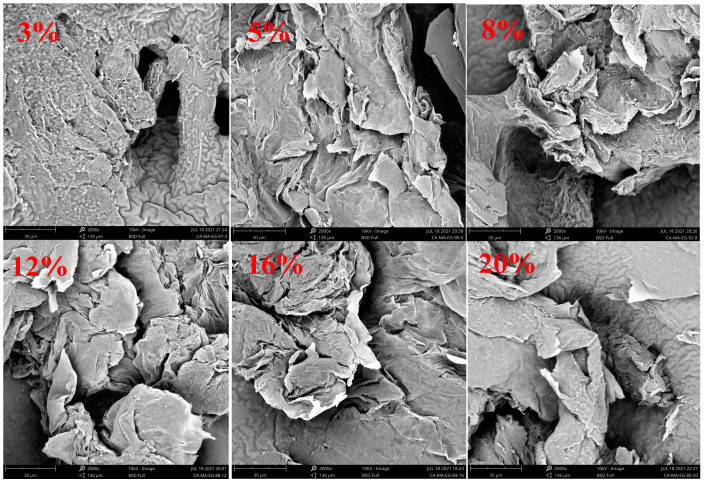
SEM pictures of fatty acid/EG CPCMs with different EG mass contents.

The CA–MA/EG CPCM samples with EG mass contents of 3%, 5%, 8%, 12%, 16%, and 20% were placed under a scanning electron microscope (SEM) to observe their microstructures. The SEM images can be seen in Figure 5. In Figure 5, it can be seen that EG is a loose and porous worm-like grid structure with a large specific surface area, making it easy for molten CA–MA to adsorb into its micro-porous structure. The network pore structure of the EG is composed of graphite flakes and a large number of irregular pores. Under the capillary force of the pores, the molten fatty acid PCM can be easily adsorbed into the microporous structure of the EG. When the EG mass content in the CPCM is low, EG adsorption becomes saturated, and its surface is covered by excess liquid fatty acids. After the fatty acids solidify, block-like fatty acid clusters form on the surface of EG, covering its worm-like structure. When the EG mass content in the CPCM is 8%, the liquid fatty acids basically fill the micro-porous structure of EG, reaching a saturated state. However, as the EG mass content in the CPCM increases to 12%, 16%, and 20%, the fatty acids uniformly and completely adsorb into the pore size of EG. The liquid fatty acids do not completely fill the micro-porous structure of EG, the CPCM still retains the original worm-like form of EG, and the molten liquid fatty acids do not leak out from EG.

### 3.4. Thermostability of the CPCMs

**Figure 6:**
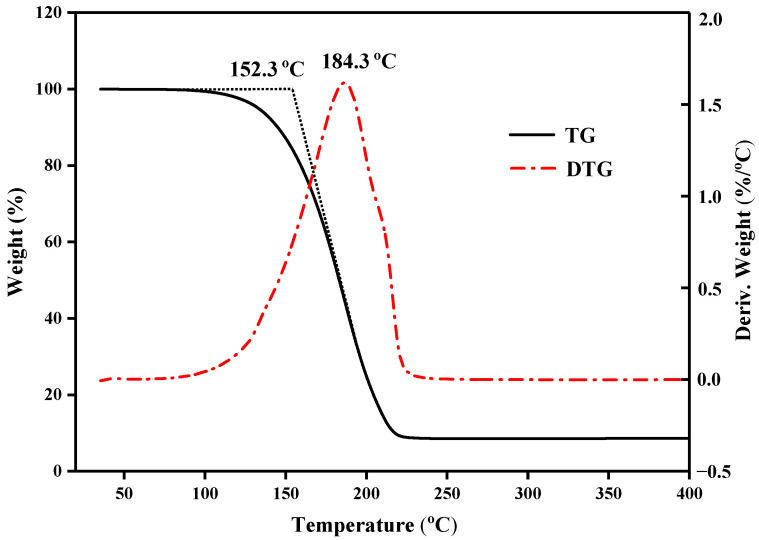
TGA curves of CA–MA/EG CPCMs.

Figure 6 displays the TGA curves of CA–MA/EG with an EG mass content of 7.6%. It is evident from this figure that, at a temperature of about 107.4 °C, CA–MA/EG started to lose weight. As the temperature increased, there was a significant loss in mass. At a temperature of roughly 205.2 °C, the PCM nearly entirely dissipated, while the weight loss rate hit its maximum at 184.3 °C. Thus, in low-temperature applications below 100 °C, CA–MA/EG composite materials exhibit good thermal stability.

### 3.5. Infrared Spectral Analysis

**Figure 7:**
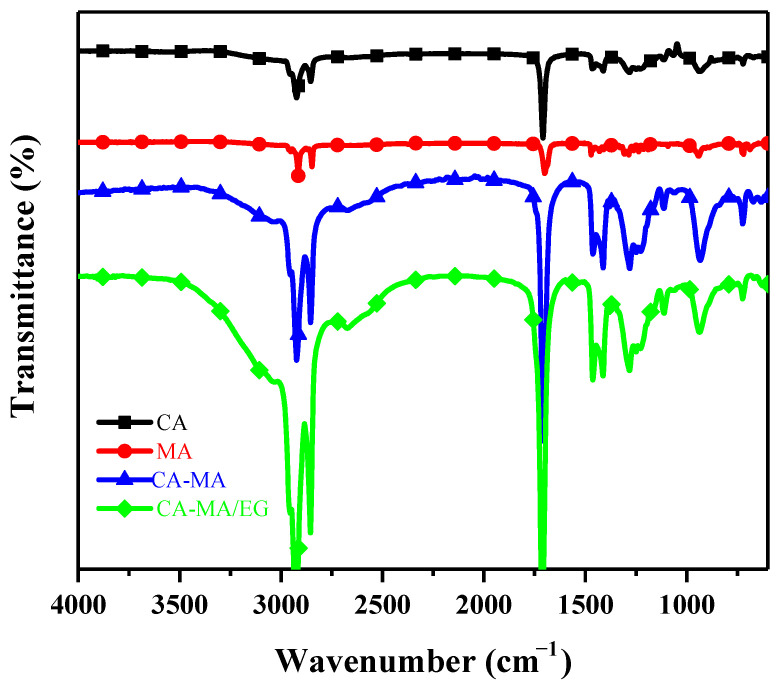
FT–IR curves of CA, MA, CA–MA, and CA–MA/EG.

CA, MA, CA–MA, and CA–MA/EG were characterized using Fourier transform infrared spectroscopy (FT–IR). Figure 7 displays the FT–IR curves. The spectra show that the C=O absorption peak is around 1708 cm^−1^, and the bending vibration peak of –CH_2_– is about 1463 cm^−1^. The absorption peak in the wavenumber range of about 2715–3105 cm^−1^ overlaps with the aliphatic group’s C–H stretching vibration absorption peak. The peak that is absorbed by the hydroxyl O–H stretching vibration is this C–H one. The spectral curves of CA, MA, CA–MA, and CA–MA/EG are comparable, and the characteristic peaks correspond one on one, according to the data for C–O above. This shows that, following the addition of expanded graphite, the structures of CA, MA, and CA–MA remained constant, and no new compounds were produced.

### 3.6. Heat Transfer Performance Experiments

**Figure 8:**
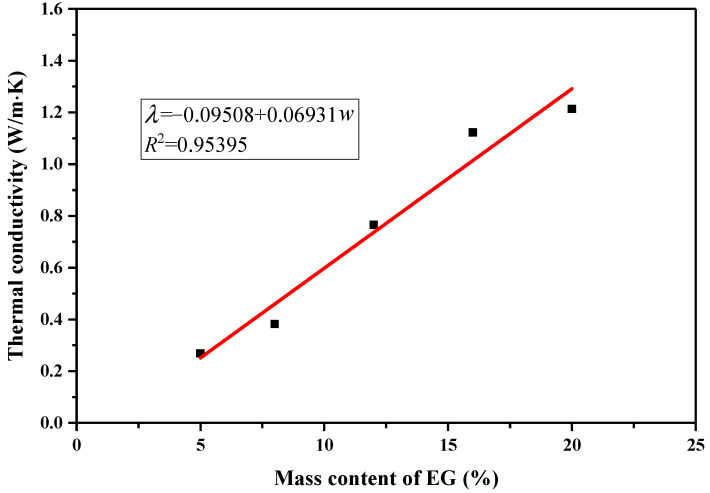
Variations in thermal conductivity of CPCMs with different EG mass contents.

The amount of EG added has a significant impact on the thermal conductivity of form-stable phase-change materials. Figure 8 shows the thermal conductivity curves of the CA–MA/EG CPCMs with EG mass contents of 5%, 8%, 12%, 16%, and 20%, and the corresponding data are listed in Table 5. When the mass content of EG in the CPCM was 1%, the CPCM was clearly a flowable liquid, and when the mass content of EG was 3%, the CPCM was a viscous fluid; therefore, the CPCMs with EG mass contents of 1% and 3% were not measured. From Figure 8, we can see that the density of the CPCMs used for measurement was 300 Kg/m^3^. The thermal conductivity of the CPCMs with EG mass contents of 5%, 8%, 12%, 16%, and 20% was 0.2683, 0.3827, 0.7656, 1.1227, and 1.2130 W/(m·k), respectively. It can be seen that the thermal conductivity of the composite material gradually increased with the increase in the EG mass content. The thermal conductivity of the CPCM with a mass content of 20% was 418.5% higher than that with a mass content of 5%. Table 4 also shows the thermal conductivity of CPCMs after adding expanded graphite to the PCMs in some of the literature, which also indicates that EG can effectively improve the heat transfer performance of fatty acid PCMs. As mentioned above, when the mass content of EG in a CPCM is low, EG is in a state of adsorption saturation, its surface is covered with excess liquid fatty acids, and heat transfer mainly occurs through convection. As the mass content of EG in a CPCM increases, liquid fatty acids gradually fill the micro-porous structure of EG until adsorption is completed, and heat transfer mainly occurs through conduction.

### 3.7. Heat Energy Storage and Release of the CPCMs

**Figure 9:**
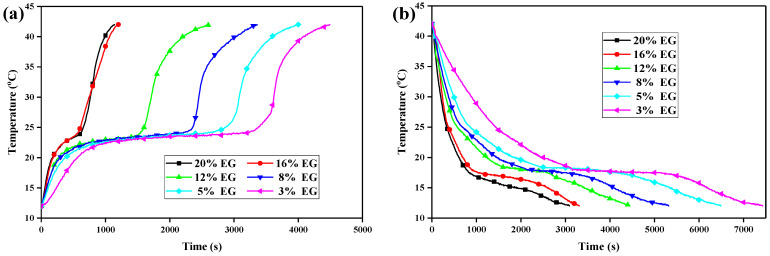
Heat energy storage and release curves of CA–MA/EG CPCMs with different EG mass contents. (**a**) Heat energy storage, (**b**) heat energy release.

In the processes of storing and releasing heat energy, the temperature settings were 42 °C and 12 °C, respectively. The test results are shown in Figure 9, with Figure 9a showing the heat energy storage curves and Figure 9b showing the heat energy release curves. In Figure 9, it can be seen that, when the EG mass contents in the CPCMs were 3%, 5%, 8%, and 12%, the melting curves had a clear “sensible heat–latent heat–sensible heat” three-stage characteristic similar to that of a pure PCM. However, as the EG mass content in the CPCM increased, the latent heat stage characteristics gradually weakened. When the EG mass contents were 16% and 20%, the latent heat stage times of the curve were extremely short, and the curve changed almost linearly. The heat energy storage times of the six sample groups were 4490 s, 4000 s, 3360 s, 2610 s, 1200 s, and 1140 s, and the heat energy release times were 7430 s, 6490 s, 5320 s, 4420 s, 3310 s, and 3090 s, respectively. From the morphological changes in the curves, it can be seen that the heat transfer law of the CPCM changed with the increase in the EG mass content. The heat transfer during the phase-change stage of the heat storage/release process was dominated by the phase-change heat transfer of the CPCM, and the pore structure of EG played an auxiliary role in enhancing the thermal conductivity. When the phase-change heat transfer characteristics of the curves disappeared, the PCM wrapped in the pores of EG became liquid, and the heat transfer of the material was dominated by the thermal conductivity of EG.

**Figure 10:**
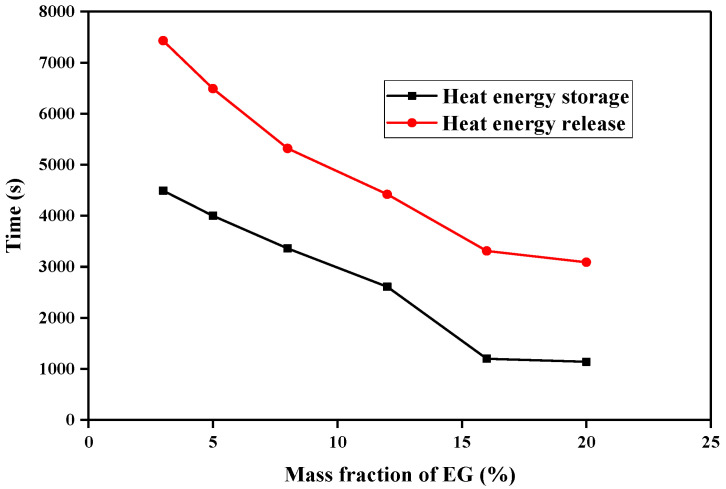
Relationship between EG content in CA–MA/EG CPCMs and heat storage/release time.

Figure 10 shows a comparison of the effect of EG on the completion time of heat storage/release in the CA–MA/EG CPCM. It can be seen that, as the EG mass content in the CPCM increases, the completion time of heat storage/release is shortened. The rate of change in the completion time of heat storage/release is the highest when the EG mass content in the CPCM increases from 3% to 15%. However, the optimization effect of increasing the EG mass content in the CPCM from 15% to 20% is not significant. Considering that leakage occurs when the EG mass content in the CPCM is less than 8%, it can be considered that 8–10% is the optimal mass content range in the CA–MA/EG CPCM for EG.

## 4. Conclusions

In this study, a series of fatty acid composite phase-change materials with different expanded graphite mass contents (1%, 3%, 5%, 8%, 12%, 16%, and 20%) were prepared by taking capric–myristic acid/expanded graphite as an example. The main conclusions drawn from the adsorption properties of expanded graphite on phase-change materials, thermal properties, microstructure, thermostability, infrared spectral analysis, heat transfer performance, and heat energy storage and release in composite phase-change materials are as follows:

(1) The minimum expanded graphite mass content in capric–myristic acid/expanded graphite composite phase-change materials is 7.6%. When the mass content of expanded graphite in composite phase-change materials exceeds the minimum content, the liquid fatty acid phase-change materials are completely filled into the porous structure of expanded graphite. Following the addition of expanded graphite, the structures of phase-change materials remain constant, and no new compounds are produced.
(2) The latent heat of composite phase-change materials decreases almost linearly with an increase in the expanded graphite mass content, and expanded graphite has almost no effect on the phase-change temperature. In low-temperature applications below 100 °C, capric–myristic acid/expanded graphite composite phase-change materials exhibit good thermal stability.
(3) The thermal conductivity of composite phase-change materials gradually increases with an increase in the expanded graphite mass content. With an increase in the expanded graphite mass content in composite phase-change materials, the heat transfer mainly transitions from phase-change heat transfer to thermal conductivity, but when the expanded graphite mass content exceeds 15%, an increase in the expanded graphite mass content has no significant effect on the composite phase-change material heat storage/release time.

The research presented above leads us to the conclusion that the prepared composite phase-change materials can be widely used in building energy-saving systems, such as floors in floor radiation heating systems, and as backfill materials for buried pipe wells in ground source heat pump systems. This will be explored by the authors in future work.

## Figures and Tables

**Table 1:** The research on graphite or other materials as supporting materials in previous works and this study.

PCM	Supporting Material	Mass Content	$T_{m}$ (°C)	$\Delta H_{m}$ (J/g)	Application	Literature
Stearic acid (SA) + benzamide (BN)	Boron nitride + expanded graphite (BG)	15 wt.% BN + 20 wt.% BG	65.21	132.35	Solar hot-water system	[38]
PA	Polyvinyl butyral + expanded graphite	3 wt.% EG	59.1	125.88	Low-temperature solar energy systems	[39]
		5 wt.% EG	58.5	124.99		
		7 wt.% EG	56.1	122.05		
Paraffin wax	Graphite	0–7 wt.%	/	/	TOS device applications	[40]
	Graphene	0.001–0.07 wt.%	/	/		
Paraffin wax	Graphite	0.007–7 wt.%	Around 52	143.03–131.35	Future networks and electric noise-free remote aerial laser switching applications	[41]
	Graphene	0.001–0.7 wt.%	59.7–52.1	134.4–108.8		
Capric acid + myristic acid	Expanded graphite	0 wt.%	19.4	150.9	Low-temperature LHTES systems and backfill materials in ground source heat pump systems	This study
		1 wt.%	18.4	148.4		
		3 wt.%	18.8	142.5		
		5 wt.%	18.5	142.8		
		8 wt.%	18.5	138.0		
		12 wt.%	18.6	129.9		
		16 wt.%	19.2	128.8		
		20 wt.%	19.2	111.8		

**Table 2:** Thermal properties of capric, lauric, myristic, palmitic, and stearic acids.

Fatty Acid	Melting		Freezing	
	$T_{m}$ (°C)	$\Delta H_{m}$ (J/g)	$T_{f}$ (°C)	$\Delta H_{f}$ (J/g)
Capric acid	31.40	169.4	31.69	170.3
Lauric acid	43.10	183.6	44.06	183.2
Myristic acid	52.68	188.6	51.63	193.1
Palmitic acid	60.60	198.1	61.10	199.3
Stearic acid	68.90	209.8	67.60	202.2

**Table 3:** CA–MA/EG composite phase-change material seepage stability assessment.

Mass Content of EG (%)	Exudation Circle Average Diameter (mm)	Leakage Percentage (%)	Assessment Standard	Assessment Result
1%	/	/	/	/
3%	88.0	193.0	Φ > 50	Extremely unstable
5%	46.5	55.0	Φ > 50	Extremely unstable
8%	0	0	Φ ≤ 0	Very stable
12%	0	0	Φ ≤ 0	Very stable
16%	0	0	Φ ≤ 0	Very stable
20%	0	0	Φ ≤ 0	Very stable

**Table 4:** Thermal properties of CA–MA/EG with different EG mass contents.

*W* (EG)%	Melting				Freezing			
	$T_{m}$ (°C)	$\Delta H_{m}$			$T_{f}$ (°C)	$\Delta H_{f}$		
		Experimental Value (J·g^−1^)	Calculated Value (J·g^−1^)	Difference (%)		Experimental Value (J·g^−1^)	Calculated Value (J·g^−1^)	Difference (%)
0	19.4	150.9	/	/	18.4	149.2	/	/
1	18.4	148.4	149.4	0.66	19.6	146.8	147.7	0.61
3	18.8	142.5	146.4	2.65	19.4	143.0	144.7	1.19
5	18.5	142.8	143.4	0.39	17.6	141.2	141.7	0.38
8	18.5	138.0	138.8	0.60	18.3	135.4	137.3	1.36
12	18.6	129.9	132.8	2.18	17.7	125.0	131.3	4.80
16	19.2	128.8	126.8	–1.61	18.0	127.1	125.3	–1.41
20	19.2	111.8	120.7	7.39	17.7	110.0	119.4	7.84

**Table 5:** Thermal conductivity of CPCMs with different EG mass contents.

CPCMs	Mass Content of EG	Thermal Conductivity (W·m^−1^·k^−1^)	Literature
LA–SA/EG	5%	0.466	[26]
	15%	0.585	
CA–LA–PA/EG	5%	0.738	[27]
CA–LA/EP/EG	5%	0.244	[28]
CA–LA/EVM/EG	5%	0.253	
LA–SA–FA–EA	3%	2.545	[29]
	5%	3.229	
	7%	6.045	
CSOD/EG	8.33%	3.136	[30]
CA–MA/EG	5%	0.2683	This study
	8%	0.3827	
	12%	0.7656	
	16%	1.1227	
	20%	1.2130	

## Data Availability

Data are contained within the article.
